# Effects of Operating and Structural Parameters on Removal of Nitric Oxide by Oxidation in a Ceramic Hollow Fiber Membrane Contactor

**DOI:** 10.3390/membranes11090704

**Published:** 2021-09-14

**Authors:** Wei Yu, Xiaoyin Li, Fangyang Yuan

**Affiliations:** 1 Jiangsu Key Laboratory of Advanced Food Manufacturing Equipment and Technology, Jiangnan University, Wuxi 214122, China; yuwei0301@jiangnan.edu.cn (W.Y.); 6200809017@stu.jiangnan.edu.cn (X.L.); 2 School of Mechanical Engineering, Jiangnan University, Wuxi 214122, China

**Keywords:** NO absorption, hollow fiber membrane contactor, mass transfer, numerical simulation

## Abstract

A numerical study on the oxidation removal of nitric oxide in a ceramic hollow fiber membrane contactor was performed. To represent the transport and absorption process, the model was created by combining multiphase, species, reaction, and porous models. The numerical results were verified by comparing them with experimental data. The tube and lumen sides both have laminar parabolic velocity distributions. The nitric oxide concentration decreases gradually from the membrane wall to axis at the cross-section except on the inner and outer sides of the membrane tube. The equivalent diffusion length was proved useful for evaluating the entrance effect. At low concentrations, the reduction efficiency was proportional to the absorbent concentration, and at large concentrations, it neared a maximum value. The reduction efficiency was positively affected by elevated operating temperature and pressure. With a gas channel width of 13 mm, the reduction flow rate achieves its maximum. The efficiency of NO reduction per area decreases as the effective membrane length increases. Increasing the operating temperature and membrane length are recommended as design priorities due to high relative enhancements. It is not recommended to improve reduction efficiency by increasing membrane tube diameter and operating pressure in design. Changing the gas flow rate, absorbent concentration and gas channel width are moderate recommended as well.

## 1. Introduction

Nitrogen oxides (NO*x*) are considered as one of the principal pollutants in exhaust gases, as they are thought to be the source of environmental issues such as acid rain, the ozone hole, and photochemical smog [1]. Different denitration technologies have been developed to get rid of the damage caused by NO*x* in industrial processes, urban wastewater treatment, transportation, et al. Among these methods, the hollow fiber membrane contactor (HFMC) is considered as a high-efficiency device that could remove or minimize NO*x* in process engineering [2,3]. As nitric oxide (NO) is difficult to absorb directly with the aqueous solution, removing NO*x* from flue gas is more challenging than removing CO_2_ or SO_2_.There is less literature on the topic of NO*x* removal than that of other acid gases.

By advanced oxidation processes (AOPs) with a liquid absorbent such as hydrogen peroxide (H_2_O_2_), NO can be oxidized to NO_2_ which can be captured in solution [4]. Ding et al. [5] proposed an oxidation-removal process capable of removing NO*x* and SO_2_ simultaneously, and they discovered that the NO*x* removal efficiency reached 80%, and was primarily dependent on the flow rate of H_2_O_2_ solution as well as the gas volume concentration. Wang et al. [6] developed a dual oxidant system composed of H_2_O_2_/S_2_O_8_^2−^ to remove NO in a bubble column reactor. The results showed that the highest NO removal efficiency of 82% at pH 11. Their subsequent investigation into NO removal using a Na_2_S_2_O_8_/CaO_2_ solution discovered that the removal efficiency first increased rapidly and then slowed [7]. Hao et al. [8] developed an AOPs system to remove SO_2_ and NO simultaneously and achieved an efficiency of 87.8% NO reduction under optimal operating conditions. The process was improved in their further studies with dual-loop absorption [9] and absorbent [10]. The NO conversion efficiency in the report was up to 96.3%. Liu et al. [11,12] investigated the ultraviolet (UV)/H_2_O_2_/NaOH process for advanced oxidation removal of NO, and the results revealed that •OH free radicals play an important role. The mass transfer rate of NO was affected by the concentration of H_2_O_2_ and NaOH, energy density per unit solution, and gas flow rate and concentration. They established an absorption rate equation to determine the mass transfer-reaction kinetics, and the NO absorption rate increases linearly with the concentration of alkaline components. Furthermore, novel processes were tested using a wet vacuum UV-spraying reactor [13], an impinging stream reactor [14], and a microwave reactor [15] and concluded that NO absorption rates can be increased by increasing the mass transfer rate in the reactors due to the fast speed of NO reactions. Dai and Deng [16] increased the operating pressure and temperature to capture CO_2_ using HMFC. The membrane wetting effect caused low capture efficiency at high pressures, according to their experimental results. Hence, membrane wetting should be avoided in order to maintain a high mass transfer rate. Li et al. [17] created a hydrophobic membrane to avoid membrane wetting even at pressures of up to 10 bar. Because of the modification of the membrane surface, the absorption process can operate at high pressure. Above all, these studies concluded that the advantages of NO reduction via H_2_O_2_ solutions are high efficiency, low initial investment and operating costs [3].

The studies presented above are mostly based on experiments and show overall NO reduction efficiency. The mass transfer process and NO gas transport in the reactors are difficult to obtain. Recently, several researchers used computational fluid dynamics (CFD) methods to discuss the AOPs for acid gas in HFMC. Using the COMSOL software, Faiz et al. [18,19,20,21,22] numerically studied the simultaneous absorption of CO_2_ and H_2_S in HFMC. Their model took into account the wetting conditions of the membrane contactors under high pressure and demonstrated good agreement with experimental results when pseudo-conditions (1–3%) were used. However, it appears that non-wetting conditions cannot accurately predict the absorption process at high pressures in the range of 10–50 bar [22]. Marjani et al. [23,24,25] modeled and simulated the CO_2_ transport through the membrane contactors with a single fiber and found that the finite element method (FEM) can describe the absorption process and complex chemical reactions in the membrane module. Ghasem [26] developed a model that took radial and axial diffusion into consideration for the simultaneous absorption/stripping process of CO_2_, and found that the removal percent of CO_2_ is directly proportional to gas loading and solvent stripping temperature. Yu et al. [27] simulated SO_2_ absorption into the water in an HFMC employing the resistance-in-series theory, partial pore wetting conditions and a chemical enhancement factor. They believed that the non-wetted assumption of the membrane pores overestimated the absorption efficiency. Furthermore, as the operating temperature rises, the efficiency of removal decreases. Chan et al. [28] studied the water condensation in pores of polytetrafluoroethylene (PTFE) hollow fiber membranes during high-pressure CO_2_ absorption around 70 °C. They found that water condensation is a possible cause of membrane wetting in the operation of membrane contactors, especially under high-temperature conditions. Lim et al. [29] numerically investigated the mass transport of one of a bundle of fibers using the ANSYS Fluent software that is based on the finite volume method (FVM). The results showed that packing density greater than 0.6 significantly increased the pressure drop. The permeate flow rate increased in direct proportion to the increase in fiber inner diameter. Qiao et al. [30] numerically analyzed the separation of the supercritical CO_2_-water mixture using an HFMC unit. The results obtained by Fluent software indicated that higher absorb efficiency can be induced by lower mixture inlet velocity and higher solution concentration. The separation efficiency could reach as high as 96.01% by ignoring the cost of the HMFC module. Tantikhajorngosol et al. [31,32] developed a 2D mathematical model for CO_2_ and H_2_S absorption at high pressures. The comparison of experimental and numerical results revealed that increasing the pressure changed the membrane wetting conditions and the gas Henry’s constant, thereby improving absorption performance. So far, numerical studies on the oxidation removal of NO in HFMC have been difficult to come by.

In this paper, we conducted a numerical study on NO absorption using H_2_O_2_ as an absorbent. Denitration was performed in a ceramic HFMC with surface modification. Kartohardjono et al. [33] conducted a similar study. They assessed NO reduction performance using 50–150 fibers in an HFMC module, but the provided values were insufficient to compare. In a previous study [34,35], HFMC was designed and experimentally tested for NO absorption. The mass transfer rates for the AOPs were found depending on the different operating parameters. However, internal transport and gas–liquid contact are still unable to be obtained. The mass transfer rates for the AOPs were discovered to vary depending on the operating parameters. Hence, a numerical study was carried out to analyze the transport phenomena with various operating and structural parameters in order to provide a theoretical basis for further improving the design of the membrane contactor.

## 2. Experimental Apparatus

**Figure 1:**
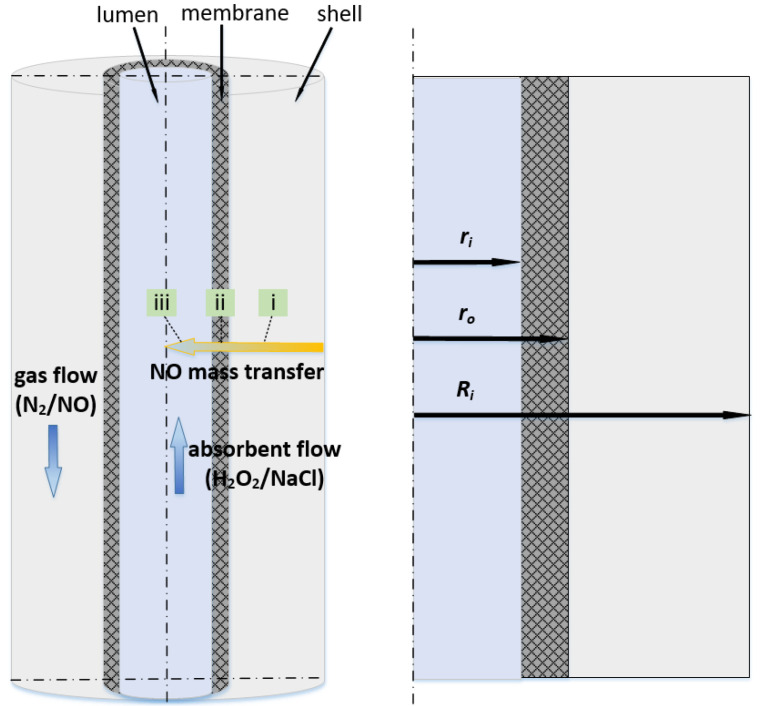
Schematic diagram of the ceramic HFMC.

Figure 1 depicts a schematic diagram of the ceramic HFMC used in this study. We discuss a single fiber characterized by a long straight cylinder. The ceramic membrane was hydrophobically modified and characterized, and the specifications of the testing membrane module are listed in our previous study [35]. It helped to achieve nonwetting conditions and over atmospheric operating conditions in order to enhance the chemical absorption of gas [36]. The size of the hollow fiber is larger than that of Kartohardjono et al. [33].

**Figure 2:**
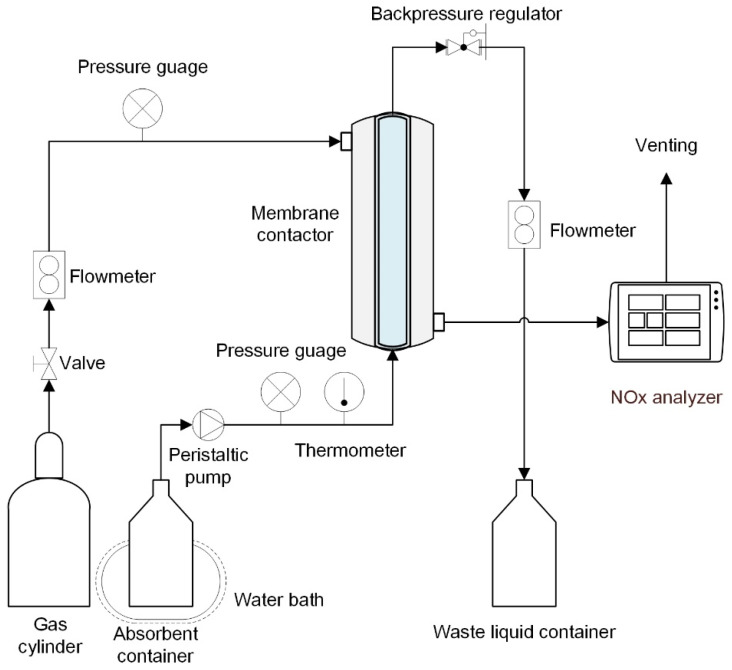
Schematic of the experimental apparatus.

Figure 2 depicts the NO absorption device utilized in the experiment. A compressed gas cylinder containing 185 ppm NO balanced with N_2_ provided the input gas. The gas pressure in the contactor during absorption can be varied by adjusting the gas cylinder’s outlet pressure. From the shell side, high-pressure gas entered the membrane module, and a mass flow meter adjusted the flow rate after the membrane. A NO concentration analyzer was used to look at the NO concentrations at the input and outflow. A gas dryer containing anhydrous calcium chloride was used to dry the gas before entering the gas analyzer to assure measurement accuracy and the safety of the electrochemical probes in the gas analyzer. The absorbent solution was fed into the SC membrane’s lumen side from a closed container, and the flow rate was controlled by a peristaltic pump and a rotational flowmeter. A pressure release valve at the outlet maintained the absorbent’s pressure. The absorbent was made by dissolving sodium chloride and H_2_O_2_ in deionized water in a specific amount. The absorbent was injected before the feed gas in the denitration process, and the absorbent pressure was slightly higher than the gas phase pressure to prevent gas bubbles from entering the liquid across the membrane. To maintain an appropriate reaction temperature, the absorbent container and SC membrane contactor were both immersed in a water bath with a specific temperature. The temperature of the absorption process was estimated by averaging the temperatures obtained by K-type thermocouples at the absorbent solution’s intake and outflow. After at least 30 min of operation, all data were gathered at steady state. For each operating state, five trials were conducted, and the results were produced by averaging the data. The gas mixture was composed of nitrogen (N_2_)/NO and was introduced into the shell of the membrane module. The absorbent solution was prepared by dissolving a certain amount of sodium chloride (NaCl) and H_2_O_2_ in deionized water and then pumped into the lumen side of the module in the opposite direction of gas flow. The hollow fiber membrane serves as a platform for NO mass transfer. The gas mixture passes through the porous area of the membrane and comes into contact with the solution. Then, there are chemical reactions:

(1) $$\mathrm{NO}+H_{2}O_{2}↔{\mathrm{NO}}_{2}+H_{2}O$$

(2) $$3{\mathrm{NO}}_{2}+H_{2}O↔2{\mathrm{HNO}}_{3}+\mathrm{NO}$$

As a result, the NO can be absorbed by the H_2_O_2_ solution, but the N_2_ is blocked.

## 3. Numerical Model

The performance of the HFMC was studied using numerical simulations. As multispecies and mass transfer occur, a coupled model to describe the gas–liquid contact should be developed. Some assumptions are made to simplify the mathematical model of the process:

- The operating process is steady and in thermodynamic equilibrium;
- The gas and liquid flows are both laminar and incompressible;
- Henry’s law applies to the gas–liquid interface.
- A non-wetted condition occurs when gas fills the pores of the membrane and liquid cannot penetrate the pores.

Based on these assumptions, the absorption process is determined by the multiphase model, species transport model, porous medium model and chemical reactions.

### 3.1. Governing Equations

As illustrated in Figure 1, a single fiber is divided into three sections, i.e., tube side, membrane, and shell side. The gas–liquid contact occurs on the membrane’s inner surface. The volume of fluid (VOF) model was implemented to determine the transport of the local volume fraction of gas and liquid phases [37,38]. The governing equations are as follows:

(3) $$\frac{\partial \rho }{\partial t}+\nabla \cdot \left(\rho \vec{U}\right)=0$$

(4) $$\frac{\partial }{\partial t}\left(\rho \vec{U}\right)+\nabla \left(\rho \vec{U}\vec{U}\right)=-\nabla P+\rho \vec{g}+\nabla \cdot \bar{\bar{T}}+\vec{F}$$

where $\vec{U}$, *P*, *ρ*, *g*, *T* and *F* are the local velocity, pressure, density, dynamic viscosity, gravity, viscous deformation tensor and capillary force. A scalar, *q*, specifies the location of each phase (volume fraction). The volume fraction for the *q* phase follows the transport equation:

(5) $$\frac{1}{\rho_{q}}\left[\frac{\partial }{\partial t}\left(\varphi_{q}\rho_{q}\right)+\nabla \left(\varphi_{q}\rho_{q}\vec{U_{q}}\right)=S_{\alpha q}+\overset{n}{\underset{p=1}{\sum }}\left({\dot{m}}_{pq}-{\dot{m}}_{qp}\right)\right]$$

The volume fractions of all phases add up to one:

(6) $$\alpha_{L}+\alpha_{G}=1$$

where ${\dot{m}}_{qp}$ is the mass transfer from phase *q* to phase *p* and ${\dot{m}}_{pq}$ is the mass transfer from phase *p* to phase *q*. The source term $S_{\alpha q}$ is zero in this work. The interface velocity is denoted by *U_q_*. The interface is located in cells where 0 < *φ_q_* < 1. Otherwise, the volume fraction function in the liquid has a value of *φ_q_* = 1. The local density and viscosity are calculated using linear interpolation as follows:

(7) $$\mu =\mu_{L}\alpha_{L}+\mu_{G}\left(1-\alpha_{L}\right)$$

(8) $$\rho =\rho_{L}\alpha_{L}+\rho_{G}\left(1-\alpha_{L}\right)$$

where the subscripts *G* and *L* stand for gas and liquid, respectively.

The solubility of chemical species at the gas–liquid interface is described by Henry’s Law. It enables the interpretation of the concentration jump at the interface as a continuous phenomenon, with the Henry solubility law converted into a solubility flux [38]. The constant of Henry’s law varies with temperature [39]:

(9) $$k_{H}\left(T\right)=k_{H}^{0}\exp\left(\frac{d\left(\ln\left(k_{H}\right)\right)}{d\left(1/T\right)}\left(\frac{1}{T}-\frac{1}{298.15}\right)\right)$$

where $k_{H}^{0}$ is Henry’s law constant for solubility in water at 298.15 K and is the temperature dependence constant.

The species transport conservation equation in the gas phase is as follows:

(10) $$\rho \nabla \cdot \left(\vec{v}Y_{q}\right)=-\nabla \cdot \vec{J_{q}}+R_{q}$$

where *Y_q_*, denotes the local mass fraction of each species; *R_q_* is the net rate of production of species *q* via chemical reaction. The diffusion flux ($\vec{J_{q}}$) in laminar flows is calculated as follows:

(11) $$\vec{J_{q}}=-\rho D_{q,m}\nabla Y_{q}-D_{T,q}\frac{\nabla T}{T}$$

where $D_{q,m}$ is the mass diffusion coefficient of species *q*, and $D_{T,q}$ is the thermal diffusion coefficient.

The porous medium model was selected to simulate the membrane zone. The effect of porous medium can be obtained by incorporating a momentum source term into the flow equation. This momentum source term is a combination of a viscous and an inertial loss term, as expressed by:

(12) $$S_{p}=-\left(\overset{3}{\underset{q=1}{\sum }}D_{pq}\mu v_{q}+\overset{3}{\underset{q=1}{\sum }}C_{pq}\frac{1}{2}\rho \left|v\right|v_{q}\right)$$

where μ is the dynamic viscosity of the fluid. The source phrase for a homogeneous porous material can be written as:

(13) $$S_{p}=-\left(\frac{\mu }{\alpha }v_{p}+C_{2}\frac{1}{2}\rho \left|v\right|v_{p}\right)$$

where α is the permeability, and *C*_2_ is the inertial resistance factor. Darcy’s Law can thus be used to compute the pressure decrease in laminar flows through a porous medium:

(14) $$\nabla p=-\frac{\mu }{\alpha }\vec{v}$$

The viscosity resistance, which is equal to 1/α, is given by the Ergun equation:

(15) $$\frac{1}{\alpha }=\frac{150{\left(1-\epsilon \right)}^{2}}{d_{p}^{2}\epsilon^{2}}$$

where ϵ is the porosity; $d_{p}$ is the equivalent diameter of membrane pores.

The Arrhenius expression is used to calculate the rates of chemical reactions in Equations (1) and (2) [1,40]:

(16) $$k_{f,r}=A_{r}T^{\beta_{r}}e^{-E_{r}/RT}$$

where *A_r_* is the pre-exponential factor, *β_r_* is the dimensionless temperature exponent, *E_r_* is the activation energy for the reaction, and *R* is the universal gas constant.

### 3.2. Numerical Methods

The computational region was axisymmetric, and the calculations were carried out on a simplified two-dimensional domain. Rectangular structured grids were used to discretize the domains, which were refined near the membrane zone. The effective membrane diameter and length of the typical membrane module used for tests in experiments is 30 and 360 mm. The membrane tube inner and outer diameters are 8 and 12 mm, respectively. The membrane tube porosity was measured as 0.38, and the average diameter of pore size is 0.2 μm.

The operating temperature was set between 25 and 70 °C, and the pressure on both sides of the membrane ranged from 1 to 6 bar. The inlet flow rates of the gas mixture and solution were 60–200 mL/min and 10–80 mL/min, respectively. Mixing theory is used to obtain the mixture parameters. The diffusivity of the gas mixture was calculated using the kinetic theory.

The simulation was carried out in parallel using ANSYS Fluent. To solve the governing equations, the coupled pressure-velocity algorithm was used. A second order scheme was used to solve the species and momentum terms.

## 4. Results and Discussion

In this paper, the effects of structure and operating parameters on the NO reduction efficiency were discussed one by one using the control variable method in the single ceramic HFMC. The NO reduction efficiency is defined as follows:

(17) $$\eta =\frac{C_{NO-in}-C_{NO-out}}{C_{NO-in}}\times 100\%$$

It should be noted that a single ceramic fiber membrane was used in this study, so the overall reduction efficiencies are not as high as those reported by Kartohardjono et al. [33]. The size of hollow fiber varies greatly, and the removal efficiency per fiber cannot be compared.

### 4.1. Flow Field and Distribution of NO Concentrations

**Figure 3:**
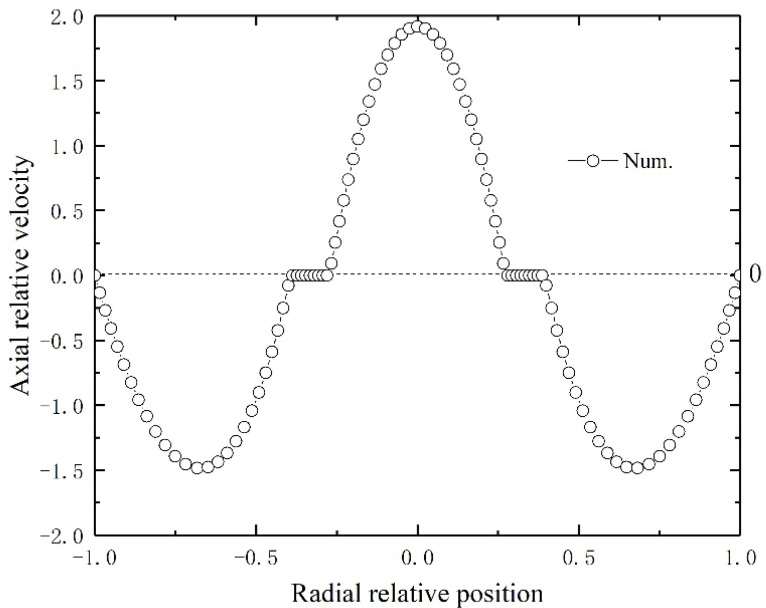
Axial relative velocity distribution in the membrane tube’s middle section.

Figure 3 depicts the countercurrent liquid and gas flow characteristics in the HFMC. For the liquid flow on the tube side and the gas flow on the lumen side, laminar parabolic velocity distributions are obtained. The relative velocity normalized by the inlet velocity of gas and liquid indicates typical laminar fluid flow in annular and tube flow channels.

**Figure 4:**
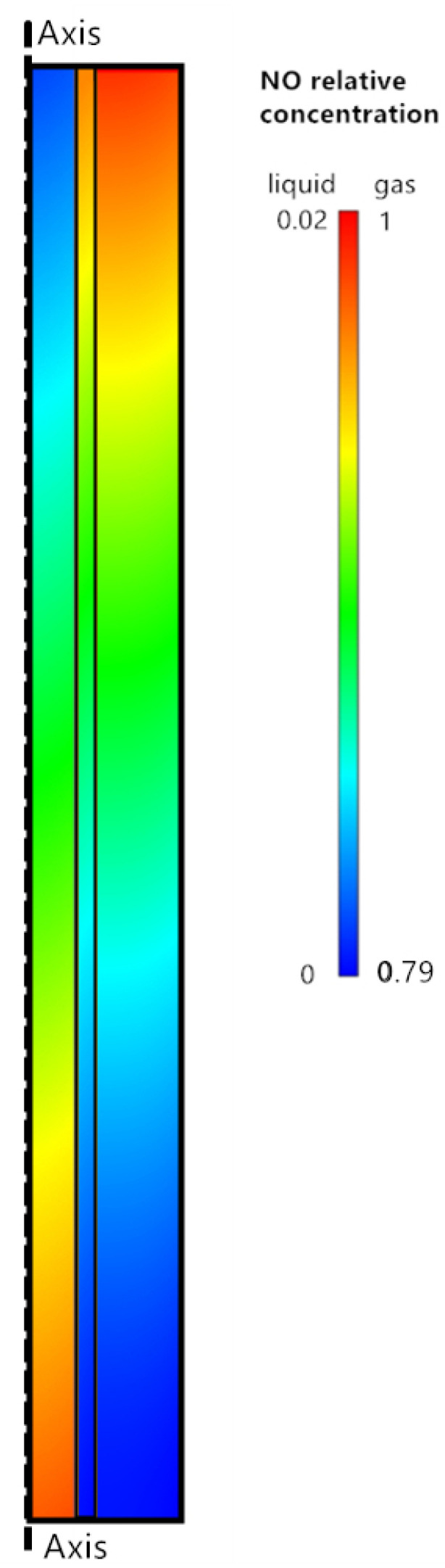
NO distribution contour along porous membrane (benchmark case parameters: feed gas of 185 ppm NO balanced with N_2_; absorbent of 0.2 wt % H_2_O_2_ and 5 wt % NaCl; absorbent flow rate of 40 mL min^−1^; absorption temperature of 343 K; ambient pressure).

Figure 4 depicts the NO distribution contour along the porous membrane. Except for the inner and outer sides of the membrane tube, the NO concentration decreases gradually from the membrane wall to the axis in a cross-section. The gas–liquid contacting interface is located on the membrane’s inner surface. When NO diffuses to the liquid surface, mass transfer slows and a large-concentration gradient forms at the surface. As a result, mass transfer resistance is primarily present in the liquid phase absorption process. The concentration of NO near the membrane surface in the liquid phase is greater than that at the axis at a given cross-section. NO diffuses slowly in the liquid phase and reacts with H2O2. These findings are consistent with those of Qazi et al. [41] and Hua et al. [42].

### 4.2. Effect of Inlet Flow Rate

To compare the diffusion length with the flow channel width, the Einstein–Smoluchowski diffusion theory induced an equivalent diffusion length *L_de_*:

(18) $$L_{de}=\frac{L_{d}}{D_{h}}=\frac{\sqrt{2DL/U}}{D_{h}}$$

where *L_d_* denotes the diffusion length perpendicular to the flow direction after a distance of fluid flow in a pipeline. *L* is the effective length of the membrane fiber and *U* is the gas mixture’s inlet velocity. *D* is the gas diffusion coefficient, and that is the molecular diffusion coefficient for the laminar flow in this study. The hydraulic diameter of the concentric annular tube flow *D_h_* = *D_i_* − *d_o_*. The value of this dimensionless parameter can be used to evaluate the entrance effect of the membrane module which refers to the development of inlet gas flow. From the formula, the entrance effect is related to gas diffusion rate, inlet velocity, hollow fiber length and gas flow channel. The Reynolds number for gas flow can be defined as Re = *UD_h_*/*υ*, in which *υ* is the viscosity of the gas mixture. Hence, the equivalent diffusion length can also be written as:

(19) $$L_{de}=\frac{L_{d}}{D_{h}}=\sqrt{\left(2\upsilon D\right)\cdot Re\cdot \left(\frac{L}{D_{h}}\right)}$$

The first part on the right of the equation represents the gas properties that are related to temperature. The Reynolds number indicates the flow velocity. The last part on the root sign of the above equation is the ratio of flow channel length and width.

**Figure 5:**
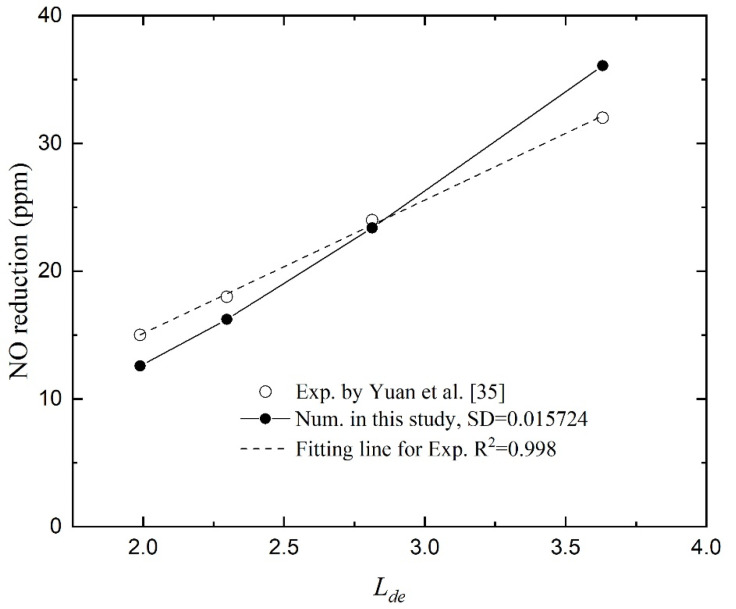
NO reduction as a function of equivalent diffusion length.

Figure 5 depicts the reduction in NO as a function of equivalent diffusion length. The numerical results indicate that the NO reduction is proportional to the equivalent diffusion length, which agrees with our previous experimental study [35]. Taking the function values obtained by linear fitting of the experimental data as the standard values, the standard deviation (SD) is 0.015724. It proves the effectiveness of the numerical model. Under the same operating conditions, the numerical results show a smaller change in NO concentration in the small *L_de_* region (gas inlet velocity is large), which indicates a greater entrance effect. Because the actual channels of the porous medium for gas diffusion are three-dimensional and complex, the radial diffusion at the developing region near the inlet may be stronger than that of the numerical simulations. While in the large *L_de_* region (gas inlet velocity is small), the numerical results present larger NO reduction than experimental data.

### 4.3. Effect of H_2_O_2_ Concentration

**Figure 6:**
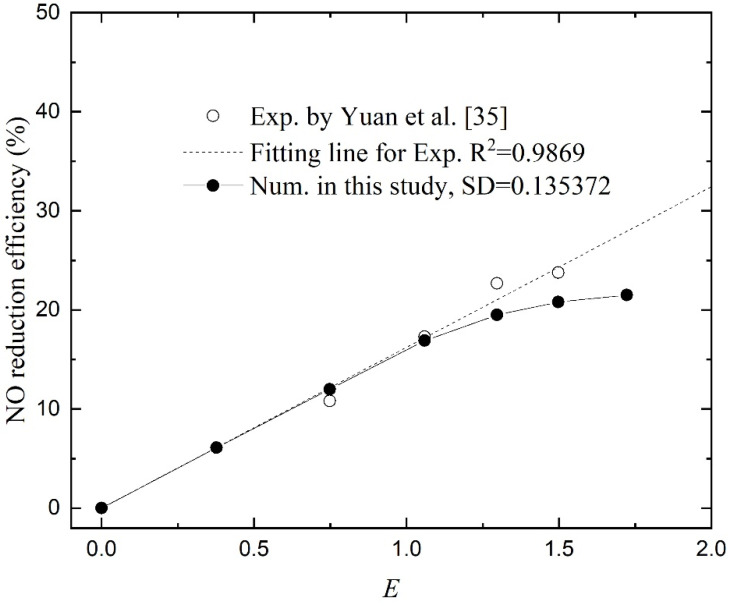
NO reduction as a function of the enhancement factor.

Yuan et al. [35] investigated the effect of H_2_O_2_ concentration on the absorption of NO. To compare the effect of chemical reaction to physical absorption, the enhancement factor *E* was used. For a high Gz number [27], the strength of chemical absorption was proportional to the square root of the H_2_O_2_ concentration. The numerical results are given in Figure 6 and the NO reduction efficiency varies by changing the H_2_O_2_ concentration in the absorbent. From the trend of the curves, the numerical results are in good agreement with the experimental data, especially in the area with a small *E*. While in the large *E* regions, the differences become more pronounced. Taking the function values obtained by linear fitting of the experimental data as the standard values, the SD is 0.135372. It also proves the effectiveness of the numerical model.When *E* < 1, the NO reduction efficiency increases linearly with the enhancement factor. At this region, the absorption rate is determined by the rate of chemical reaction, which is subject to the H_2_O_2_ concentration at the reaction site. When *E* > 1, the H_2_O_2_ concentration can support abundant chemical reactions. The entire process is constrained by the rate of gas diffusion and gas–liquid contact. In this case, the change of NO reduction slows down with the increase in the enhancement factor, and the removal efficiency appears to approach a maximum value. As shown in Figure 1, the mass transfer of NO process consists of three consecutive steps: (i) diffusion from the bulk gas phase to the membrane’s outer surface; (ii) diffusion through the membrane pores to the gas–liquid interface; and (iii) dissolution into the absorption liquid and liquid-phase diffusion/chemical reaction. The resistance in series model can be used to describe the entire process [43,44,45]:

(20) $$k_{o}^{-1}={\left(k_{g}\cdot \frac{d_{o}}{d_{i}}\right)}^{-1}+{\left(k_{m}\cdot \frac{d_{lm}}{d_{i}}\right)}^{-1}+H{\left(E\cdot k_{l}\right)}^{-1}$$

Our previous study used only five points to present the data, and the flattening trend was not concluded, but it can be seen from the rightmost two points obtained through experiments.

### 4.4. Effect of Operating Temperature

**Figure 7:**
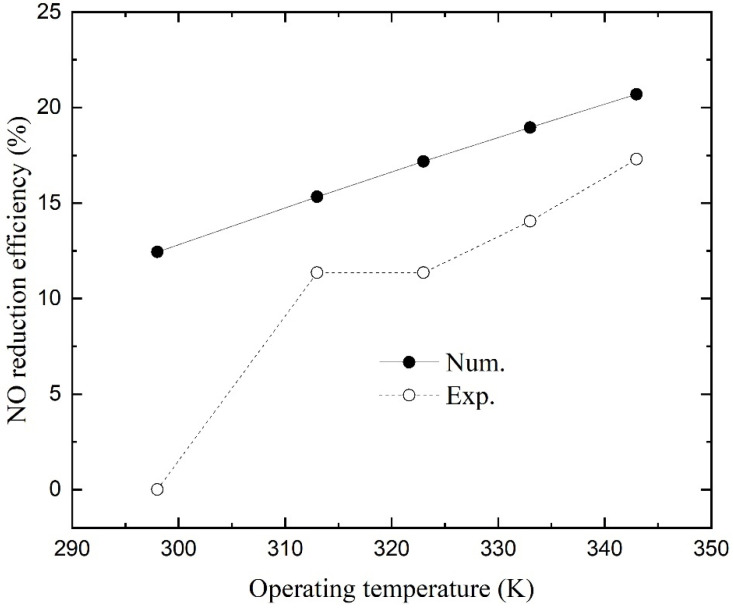
NO reduction efficiency as a function of operating temperature.

Figure 7 shows the numerical and experimental NO reduction efficiencies as a function of operating temperature. The numerical simulations presented higher efficiencies than those of experiments, and both show that temperature has a positive effect on NO absorption. The efficiency increases linearly with operating temperature, and the second point obtained from experiments when the temperature was 313.15 K in the previous study may have been recorded incorrectly (its value is similar to that of the latter point at *T* = 323.15 K). On the one hand, the effect of temperature on efficiency is caused by an increase in the gas diffusion coefficient. According to the Maxwell–Gilliland formula, gas diffusivities increase with increasing temperature:

(21) $$D=D_{0}\left(\frac{p}{p_{0}}\right){\left(\frac{T}{T_{0}}\right)}^{3/2}$$

where *D*_0_ is the diffusion coefficient for a certain temperature *T*_0_ and pressure *P*_0_. The NO diffusion coefficient is proportional to *T*^3/2^. Besides, the increase in temperature induces an increase in NO permeability through the membrane [46]. Hence, the first two terms on the right hand of Equation (20) increase with operating temperature. The reaction rate, on the other hand, will increase as the temperature rises. However, the increase in temperature will reduce the solubility of NO according to Equation (9). Under the combined action of many factors, the total mass transfer rate still keeps increasing, which reflects that an appropriate increase in operating temperature can improve NO reduction efficiency.

### 4.5. Effect of Operating Pressure

**Figure 8:**
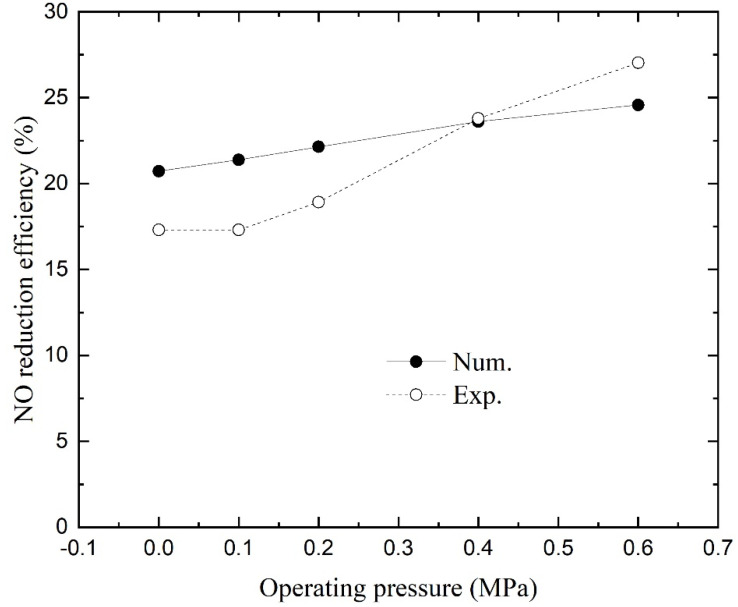
NO reduction efficiency as a function of operating pressure.

Figure 8 shows the numerical and experimental NO reduction efficiencies as a function of operating pressure. With the increase in operating pressure, the NO reduction efficiencies become larger linearly according to the numerical results. The pressure-induced change in efficiencies is flatter than the experimental data. The NO reduction was enhanced by the escalating driving force across the membrane due to the increasing NO partial pressure above the liquid [16,46]. Despite the fact that the molecular diffusion co-efficient decreases with increasing pressure according to Equation (21), the overall effect of pressure is beneficial for NO reduction. Because of the existence of transmembrane pressure, the pressure cannot be raised indefinitely. Once the membrane is partially wet, the mass transfer efficiency will be greatly reduced [27].

### 4.6. Effect of Flow Channel Width

**Figure 9:**
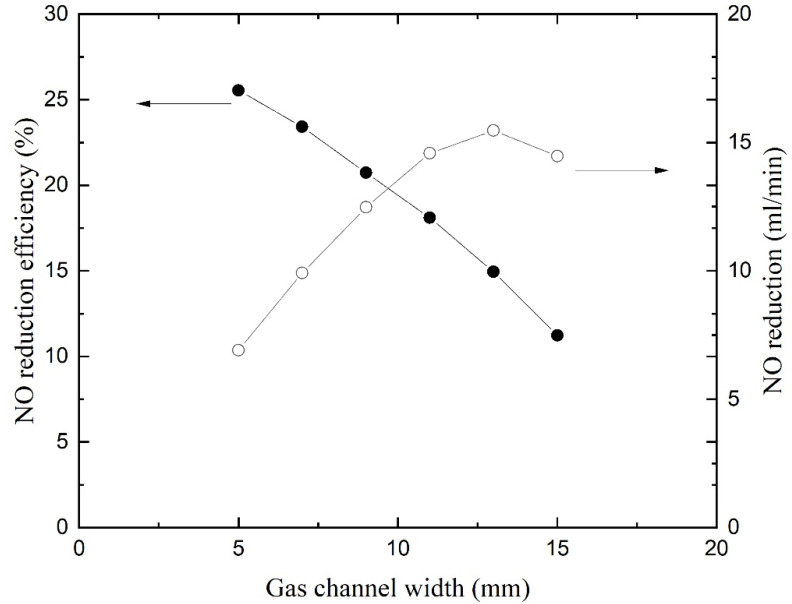
NO reduction and efficiency as a function of gas channel width.

Figure 9 gives the NO reduction and efficiency as a function of gas channel width. The curves were obtained by varying the width of gas channel and while keeping the inlet gas velocity constant. The reduction efficiency reduces with the increase in gas channel width. When the gas channel is wider, NO cannot diffuse to the liquid surface and flow out of the channel because it is too far away from the membrane. Nevertheless, the flow rate is reduced with a narrow annular channel while the inlet velocity remains constant. The NO reduction flow rates are calculated by multiplying the flow rates and reduction efficiency and are displayed on the right *y*-axis. The curve reveals that the NO reductions increase first and then decrease with the increase in gas channel width. The reduction flow rate reaches a maximum with a gas channel width of 13 mm. If the absorption process is carried out circularly in the HFMC, the total process is the fastest in this configuration. Hence, an appropriate gas channel width should be designed to achieve a good balance between one-way reduction efficiency and treatment capacity. The gas channel width of the test membrane module here is 9 mm, which is suitable.

Absorption is affected by the width of the liquid channel. The wider the channel is, the larger the NO reduction is. The enhancement of absorption is not so significant because the mass transfer rate is restricted by the gas–liquid contact. Absorbent is abundant in large liquid flow channels, which provide ample absorbent for chemical reactions. However, a wide channel leads to a larger volume of HFM and needs more absorbent flowing inside. If the membrane module is made up of hundreds of membranes, the cost will increase significantly. Besides, the strength of the membrane also needs to be considered. Hence, it is not recommended to design the solution flow channel too large.

### 4.7. Effect of Flow Channel Length

**Figure 10:**
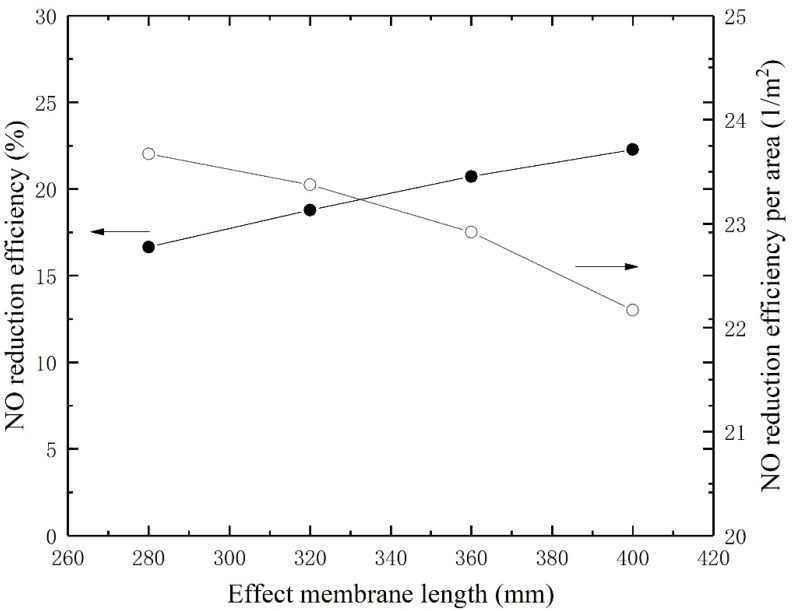
NO reduction and efficiency as a function of fiber length.

Figure 10 gives the NO reduction and efficiency as a function of fiber length. The reduction efficiency increases with the increase in effective membrane length. The mass transfer flux will be greater when the membrane is longer. However, the change of increase becomes smaller due to the smaller concentration gradient. The NO reduction efficiencies per area are calculated by dividing the efficiencies by membrane surface area (gas–liquid contacting surface) and depicted on the right *y*-axis. The curve reveals that the NO reduction efficiencies per area decrease with the increase in effective membrane length. In this study, the cost of the membrane is affected by the preparation processes such as surface modification. Hence, the membrane length should be designed to strike a balance between preparation cost and removal efficiency.

### 4.8. Investigated Parameters Significance on NO Reduction Efficiency

Effects of seven investigated parameters (*Pm*) on NO reduction efficiency have been discussed above. Here, we define an index called relative enhancement (RE) to compare the significance of investigated parameters on NO reduction efficiency:

(22) $$\mathrm{RE}=\frac{\eta_{\max}/\eta_{\min}}{Pm_{\max}/Pm_{\min}}$$

The index represents the proportion of parameters that need to be changed for equivalent improvement in NO reduction efficiency. Table 1 lists the relative enhancement and assessment of investigated parameters in descending order. The maximum and minimum values of efficiencies and parameters in Equation (18) are limited to numerical results as shown in Figure 5, Figure 6, Figure 7, Figure 8, Figure 9 and Figure 10. In Figure 6, the (0,0) point is ignored to obtain a RE for H_2_O_2_ concentration. The RE for operating pressure is calculated by absolute pressure. It is noted that the increases in NO reduction efficiency are achieved by decreasing the gas flow rate and gas channel width (Indicated by downward arrows in the table).

The maximum relative enhancement is 1.4455 by elevated operating temperature. Correspondingly, the minimum relative enhancement is 0.1694 by elevated operating pressure. Enlarging the liquid channel (lumen side) by increasing the liquid channel width leads to a small RE as well. The RE for membrane length, gas flow rate, H_2_O_2_ concentration, and gas channel width are close and range from 0.9365 to 0.7888. The cost of changing operating and structural parameters on the removal of nitric oxide also needs to be considered when designing the absorption process. Increasing the temperature leads to an increase in energy consumption, which can be achieved by a water bath, etc. Increasing the membrane length and liquid channel width leads to an increase in device size and cost. Decreasing the gas channel width can be achieved by arranging the hollow fiber membranes in parallel, which can keep the device compact and reduce device costs. Reducing the gas flow rate leads to an increase in operation time. Increasing the H_2_O_2_ concentration leads to an increase in absorbent consumption. Overall, changing the operating temperature and membrane length should be prioritized when designing the process of NO absorption. Changing the gas flow rate, absorbent concentration and gas channel width are recommended to enhancement the NO reduction as well. However, changing the liquid channel width and operating pressure are not recommended to enhancement the NO reduction due to their poor effects and high costs.

## 5. Conclusions

In this paper, a numerical study on the oxidation removal of nitric oxide in a ceramic hollow fiber membrane contactor was performed. To represent the transport and absorption process, the model was created by combining multiphase, species, reaction, and porous models. The numerical results were verified by comparing them with experimental data. The tube and lumen sides both have laminar parabolic velocity distributions. The nitric oxide concentration decreases gradually from the membrane wall to axis at the cross-section except on the inner and outer sides of the membrane tube. The equivalent diffusion length was proved useful for evaluating the entrance effect. At low concentrations, the reduction efficiency was proportional to the absorbent concentration, and at large concentrations, it neared a maximum value. The reduction efficiency was positively affected by elevated operating temperature and pressure. With a gas channel width of 13 mm, the reduction flow rate achieves its maximum. The efficiency of NO reduction per area decreases as the effective membrane length increases. Increasing the operating temperature and membrane length are recommended as design priorities due to high relative enhancements. It is not recommended to improve reduction efficiency by increasing membrane tube diameter and operating pressure in design. Changing the gas flow rate, absorbent concentration and gas channel width are moderate recommended as well.

## Figures and Tables

**Table 1:** Relative enhancement and assessment of investigated parameters on NO reduction efficiency (listed in descending order).

Investigated Parameters	RE	Cost	Overall Assessment
Operating temperature (↑)	1.445563585	Increase energy consumption	Recommended
Membrane length (↑)	0.936486486	Increase device size and cost	Recommended
Gas flow rate (↓)	0.860294118	Increase operation time	Moderate recommended
H_2_O_2_ concentration (↑)	0.771438783	Increase absorbent consumption	Moderate recommended
Gas channel width (↓)	0.758764112	Decrease device size and cost	Moderate recommended
Liquid channel width (↑)	0.337510481	Increase device size and cost	Not recommended
Operating pressure (↑)	0.169388247	Increase membrane wetting and energy consumption	Not recommended

## Data Availability

Not applicable.

## References

[1] Chen R., Zhang T., Guo Y., Wang J., Wei J., Yu Q. (2021). Recent advances in simultaneous removal of SO_2_ and NOx from exhaust gases: Removal process, mechanism and kinetics. Chem. Eng. J..

[2] Noriega-Hevia G., Serralta J., Borrás L., Seco A., Ferrer J. (2020). Nitrogen recovery using a membrane contactor: Modelling nitrogen and pH evolution. J. Environ. Chem. Eng..

[3] Gholami F., Tomas M., Gholami Z., Vakili M. (2020). Technologies for the nitrogen oxides reduction from flue gas: A review. Sci. Total Environ..

[4] Liu Y., Shi S., Wang Y. (2021). Removal of pollutants from gas streams using Fenton (-like)-based oxidation systems: A review. J. Hazard. Mater..

[5] Ding J., Zhong Q., Zhang S., Song F., Bu Y. (2014). Simultaneous removal of NOX and SO_2_ from coal-fired flue gas by catalytic oxidation-removal process with H2O2. Chem. Eng. J..

[6] Wang Z., Wang Z., Ye Y., Chen N., Li H. (2016). Study on the removal of nitric oxide (NO) by dual oxidant (H2O2/S2O82−) system. Chem. Eng. Sci..

[7] Wang Z., Zhang Y., Tan Z., Li Q. (2018). A wet process for oxidation-absorption of nitric oxide by persulfate/calcium peroxide. Chem. Eng. J..

[8] Hao R., Zhao Y., Yuan B., Zhou S., Yang S. (2016). Establishment of a novel advanced oxidation process for economical and effective removal of SO_2_ and NO. J. Hazard. Mater..

[9] Hao R., Wang X., Zhao X., Xu M., Zhao Y., Mao X., Yuan B., Zhang Y., Gao K. (2018). A novel integrated method of vapor oxidation with dual absorption for simultaneous removal of SO_2_ and NO: Feasibility and prospect. Chem. Eng. J..

[10] Hao R., Mao Y., Mao X., Wang Z., Gong Y., Zhang Z., Zhao Y. (2019). Cooperative removal of SO_2_ and NO by using a method of UV-heat/H2O2 oxidation combined with NH4OH-(NH4)2SO3 dual-area absorption. Chem. Eng. J..

[11] Liu Y., Pan J., Tang A., Wang Q. (2013). A study on mass transfer–reaction kinetics of NO absorption by using UV/H2O2/NaOH process. Fuel.

[12] Liu Y., Wang Q., Yin Y., Pan J., Zhang J. (2014). Advanced oxidation removal of NO and SO_2_ from flue gas by using ultraviolet/H_2_O_2_/NaOH process. Chem. Eng. Res. Des..

[13] Liu Y., Wang Q., Pan J. (2016). Novel process of simultaneous removal of nitric oxide and sulfur dioxide using a vacuum ultraviolet (VUV)-activated O2/H2O/H2O2 system in a wet VUV–spraying reactor. Environ. Sci. Technol..

[14] Liu Y., Wang Y. (2017). Simultaneous removal of NO and SO_2_ using aqueous peroxymonosulfate with coactivation of Cu2+/Fe3+ and high temperature. AICHE J..

[15] Wang Y., Liu Y., Shi S. (2020). Removal of nitric oxide from flue gas using novel microwave-activated double oxidants system. Chem. Eng. J..

[16] Dai Z., Deng L. (2016). Membrane absorption using ionic liquid for pre-combustion CO_2_ capture at elevated pressure and temperature. Int. J. Greenh. Gas Control.

[17] Li Y., Wang L.a., Hu X., Jin P., Song X. (2018). Surface modification to produce superhydrophobic hollow fiber membrane contactor to avoid membrane wetting for biogas purification under pressurized conditions. Sep. Purif. Technol..

[18] Al-Marzouqi M.H., El-Naas M.H., Marzouk S.A.M., Al-Zarooni M.A., Abdullatif N., Faiz R. (2008). Modeling of CO_2_ absorption in membrane contactors. Sep. Purif. Technol..

[19] Faiz R., Al-Marzouqi M. (2009). Mathematical modeling for the simultaneous absorption of CO_2_ and H2S using MEA in hollow fiber membrane contactors. J. Membr. Sci..

[20] Faiz R., Al-Marzouqi M. (2010). CO_2_ removal from natural gas at high pressure using membrane contactors: Model validation and membrane parametric studies. J. Membr. Sci..

[21] Faiz R., Al-Marzouqi M. (2011). Insights on natural gas purification: Simultaneous absorption of CO_2_ and H2S using membrane contactors. Sep. Purif. Technol..

[22] Faiz R., Li K., Al-Marzouqi M. (2014). H2S absorption at high pressure using hollow fibre membrane contactors. Eng. Process. Process Intensif..

[23] Sohrabi M.R., Marjani A., Moradi S., Davallo M., Shirazian S. (2011). Mathematical modeling and numerical simulation of CO_2_ transport through hollow-fiber membranes. Appl. Math. Model..

[24] Shirazian S., Marjani A., Rezakazemi M. (2012). Separation of CO_2_ by single and mixed aqueous amine solvents in membrane contactors: Fluid flow and mass transfer modeling. Eng. Comput..

[25] Ghadiri M., Marjani A., Shirazian S. (2013). Mathematical modeling and simulation of CO_2_ stripping from monoethanolamine solution using nano porous membrane contactors. Int. J. Greenh. Gas Control.

[26] Ghasem N. (2020). Modeling and simulation of the simultaneous absorption/stripping of CO_2_ with potassium glycinate solution in membrane contactor. Membranes.

[27] Yu H., Thé J., Tan Z., Feng X. (2016). Modeling SO_2_ absorption into water accompanied with reversible reaction in a hollow fiber membrane contactor. Chem. Eng. Sci..

[28] Chan Z.P., Li L., Kang G., Ab Manan N., Cao Y., Wang T. (2020). Discussion on water condensation in membrane pores during CO_2_ absorption at high temperature. Membranes.

[29] Lim K.B., Wang P.C., An H., Yu S.C.M. (2017). Computational studies for the design parameters of hollow fibre membrane modules. J. Membr. Sci..

[30] Qiao Z., Cao Y., Tang Y., Si F. (2020). Numerical analysis of membrane–absorption separation for supercritical carbon dioxide and water mixture of plume geothermal power generation systems. Energy Convers. Manage..

[31] Tantikhajorngosol P., Laosiripojana N., Jiraratananon R., Assabumrungrat S. (2017). Analytical study of membrane wetting at high operating pressure for physical absorption of CO_2_ using hollow fiber membrane contactors. Chem. Eng. Res. Des..

[32] Tantikhajorngosol P., Laosiripojana N., Jiraratananon R., Assabumrungrat S. (2019). Physical absorption of CO_2_ and H2S from synthetic biogas at elevated pressures using hollow fiber membrane contactors: The effects of Henry’s constants and gas diffusivities. Int. J. Heat Mass Transf..

[33] Kartohardjono S., Merry C., Rizky M.S., Pratita C.C. (2019). Nitrogen oxide reduction through absorbent solutions containing nitric acid and hydrogen peroxide in hollow fiber membrane modules. Heliyon.

[34] Fang Z., Yu X., Tang W., Yu X., Zhao S., Tu S.-T. (2017). Denitration by oxidation-absorption with polypropylene hollow fiber membrane contactor. Appl. Energy.

[35] Yuan F., Yan J., Yu W., Du J., Yu J. (2020). Nitric oxide reduction by hydrogen peroxide absorption through a ceramic hollow fiber membrane contactor. J. Environ. Chem. Eng..

[36] Ghasem N. (2019). Chemical absorption of CO_2_ enhanced by nanoparticles using a membrane contactor: Modeling and simulation. Membranes.

[37] Haroun Y., Raynal L., Alix P. (2014). Prediction of effective area and liquid hold-up in structured packings by CFD. Chem. Eng. Res. Des..

[38] Haroun Y., Raynal L. (2016). Use of computational fluid dynamics for absorption packed column design. Oil Gas Sci. Technol.—Rev. IFP Energ. Nouv..

[39] Sander R. (2015). Compilation of Henry’s law constants (version 4.0) for water as solvent. Atmos. Chem. Phys..

[40] Vadillo J.M., Gómez-Coma L., Garea A., Irabien A. (2020). CO_2_ desorption performance from imidazolium ionic liquids by membrane vacuum regeneration technology. Membranes.

[41] Qazi S., Gómez-Coma L., Albo J., Druon-Bocquet S., Irabien A., Sanchez-Marcano J. (2020). CO_2_ capture in a hollow fiber membrane contactor coupled with ionic liquid: Influence of membrane wetting and process parameters. Sep. Purif. Technol..

[42] Hua C.-G., Kang G.-D., Jia J.-X., Li M., Cao Y.-M., Yuan Q. (2013). Physical absorption of CO_2_ using polyvinylidene fluoride membrane contactor at high pressure and mathematical simulation. Chem. J. Chin. Univ..

[43] Mansourizadeh A., Ismail A.F. (2009). Hollow fiber gas–liquid membrane contactors for acid gas capture: A review. J. Hazard. Mater..

[44] Xu Y., Goh K., Wang R., Bae T.-H. (2019). A review on polymer-based membranes for gas-liquid membrane contacting processes: Current challenges and future direction. Sep. Purif. Technol..

[45] Wang Y., Yu X. Removal of NO research in A polypropylene hollow fiber membrane contactor. Proceedings of the Proceedings of the 2017 6th International Conference on Energy, Environment and Sustainable Development.

[46] Bazhenov S.D., Bildyukevich A.V., Volkov A.V. (2018). Gas-liquid hollow fiber membrane contactors for different applications. Fibers.

